# Functional Group Effects on the HOMO–LUMO Gap of g-C_3_N_4_

**DOI:** 10.3390/nano8080589

**Published:** 2018-08-03

**Authors:** Hao Li, Zhien Zhang, Yulu Liu, Wanglai Cen, Xubiao Luo

**Affiliations:** 1 College of Chemistry, Sichuan University, Chengdu 610064, China; 2 Key Laboratory of Jiangxi Province for Persistant Pollutants Control and Resources Recycle, Nanchang Hangkong University, Nanchang 330063, China; luoxubiao@126.com; 3 College of Architecture and Environment, Sichuan University, Chengdu 610064, China; yulu5412@126.com; 4 Institute of New Energy and Low Carbon Technology, Sichuan University, Chengdu 610207, China; cenwl@utexas.edu

**Keywords:** functional group, graphitic carbon nitride (g-C_3_N_4_), HOMO–LUMO gap

## Abstract

Graphitic carbon nitride (g-C_3_N_4_) is a promising semiconductor material which has been widely studied in nanoscience. However, the effect of modifying the performance of g-C_3_N_4_ is still under debate. In this communication, we show the size and functional group effects on the g-C_3_N_4_ using density functional theory (DFT) calculations. It was found that a molecule with six repeated g-C_3_N_4_ units (g-C_3_N_4_-6) could be the smallest unit that converges to the limit of its HOMO–LUMO gap. Calculations of g-C_3_N_4_-6 with varying numbers of substituted C≡N, C=O, and O−H functional groups show that C≡N and C=O could narrow down the HOMO–LUMO gap, while O−H could slightly raise the gap. This study shows that the change of substituents could tune the band gap of g-C_3_N_4_, suggesting that rationally modifying the substituent at the edge of g-C_3_N_4_-based materials could help to significantly increase the photocatalytic properties of a metal-free g-C_3_N_4_.

## 1. Introduction

Graphitic carbon nitride (g-C_3_N_4_) is a promising metal-free polymeric n-type semiconductor which has attracted huge interest during the past decade [1,2,3,4]. With its important electric, optical, structural, thermal, and chemical properties, g-C_3_N_4_ has been widely applied to electro- and photo-chemistries. Since the primary works done by Wang et al. [5], which showed that g-C_3_N_4_ is a promising photocatalyst for hydrogen evolution under visible light, g-C_3_N_4_ has been widely studied as a cost-effective photocatalyst for many reactions, such as carbon dioxide reduction [6,7,8] and photodegradation [1,9,10,11]. From experimental measurements, the band gap of g-C_3_N_4_ is usually between 2–3 eV, which could enable it to harvest sunlight with a wavelength of around 460 nm [1,6]. However, this still deviates from the well-known ideal band gap of a semiconductor (around 2.0 eV). Therefore, slightly narrowing down the band gap of g-C_3_N_4_ would be a particularly challenging but important target in the material’s modification. 

To narrow down the band gap of g-C_3_N_4_-based materials, doping with transition metal ions has been proven as an efficient strategy (e.g., cave [12,13,14] and interlayer [15] dopings). However, such a method involves a transition metal as the experimental input, which could raise the cost for industrial applications. Therefore, metal-free band gap engineering is particularly important. Currently, it is not well-known as to whether some of the modified g-C_3_N_4_-like materials could perform enhanced photocatalytic activities compared to pure g-C_3_N_4_. A better understanding of the mechanisms of band gap tuning would be beneficial to the future design and understanding of high-performance modified g-C_3_N_4_ materials.

In this paper, we examine how the HOMO−LUMO gap changes with the g-C_3_N_4_ size and correlates with the substituted functional group using density functional theory (DFT) calculations. The functional group effect on g-C_3_N_4_ with different substituted functional groups was studied, and the HOMO−LUMO gaps of g-C_3_N_4_ with varying numbers of C≡N, C=O, and O−H groups were calculated. For the first time, we found that g-C_3_N_4_ with a specific amount of substituted C≡N or C=O could narrow down the HOMO−LUMO gap; a finding which could impart significant guidance to g-C_3_N_4_ band gap engineering. 

## 2. Computational Method

All the DFT calculations were performed to calculate the HOMO−LUMO gap with the Vienna Ab initio simulation package (VASP) [16]. Electron-core interactions were described within the projector-augmented wave (PAW) method [17]. Generalized-gradient approximation (GGA) with the Perdew–Burke–Ernzerhof (PBE) functional was performed for electron exchange and correlation [18]. Kohn–Sham orbitals were expanded on a plane-wave basis [19]. The kinetic energy cutoff was set as 400 eV for all the calculations. All the configurations were considered optimal when all the forces on each atom were lower than 0.05 eV/Å. The Brillouin zone was sampled by Γ-point. The vacuum of at least 10 Å was set in the z-dimension. The lengths of the x- and y-dimensions ranged from 20 to 40 Å for the g-C_3_N_4_ structures with varying size. Convergence tests with higher kinetic energy cutoff and lower forces were performed; no significant change was found in the results. 

## 3. Results and Discussion

**Figure 1:**
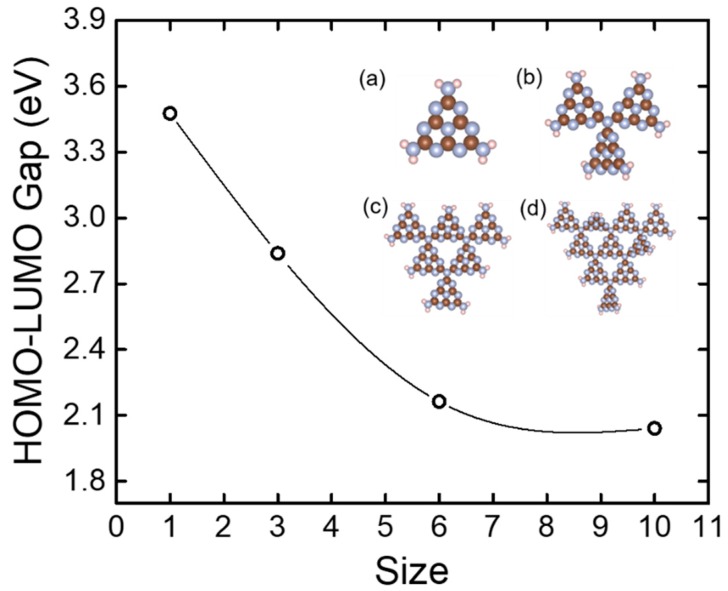
Calculated HOMO–LUMO gap vs g-C_3_N_4_ structures with varying sizes. Insets show the optimized configurations of (**a**) g-C_3_N_4_-1; (**b**) g-C_3_N_4_-3; (**c**) g-C_3_N_4_-6; and (**d**) g-C_3_N_4_-10. Brown, blue, and pink spheres represent C, N, and H, respectively.

Here, we performed DFT calculations to elucidate the effects of size and functional groups on the HOMO−LUMO gap of g-C_3_N_4_ structures. We first studied the size effect on the pure g-C_3_N_4_. Figure 1 shows that the HOMO−LUMO gap monotonically decreases with the increase of g-C_3_N_4_ repeated units and then reaches a plateau, suggesting that if the size is sufficiently large, the HOMO–LUMO gap becomes less sensitive to size. This is quite similar to a previous theoretical study on nanographene structures by Jiang and Dai [20]: there should be a critical size that leads to a convergence of the HOMO−LUMO gap of graphene or graphene-like materials. 

**Figure 2:**
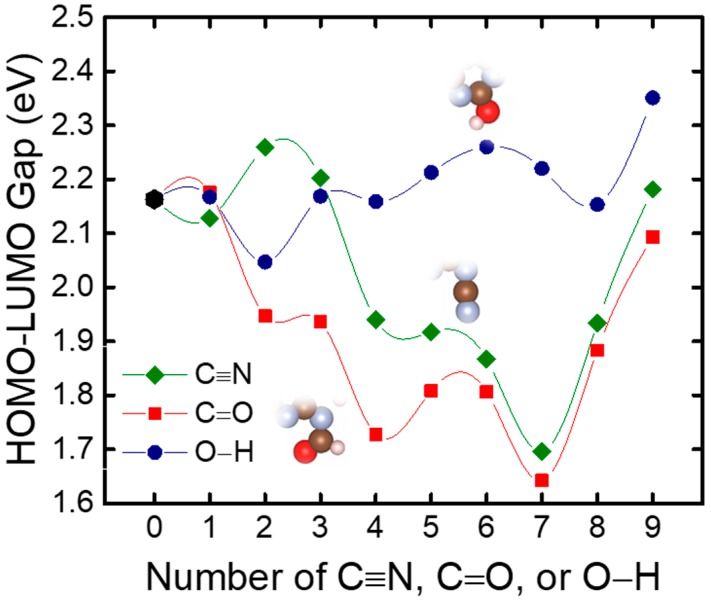
Calculated HOMO–LUMO gap vs a g-C_3_N_4_-6 structure with varying numbers of C≡N, C=O, and O−H. Configurations used for DFT calculations are shown in Figure 3, Figure 4 and Figure 5. Insets show the functional groups in the calculated configurations. Brown, blue, pink, and red spheres represent C, N, H, and O, respectively.

**Figure 3:**
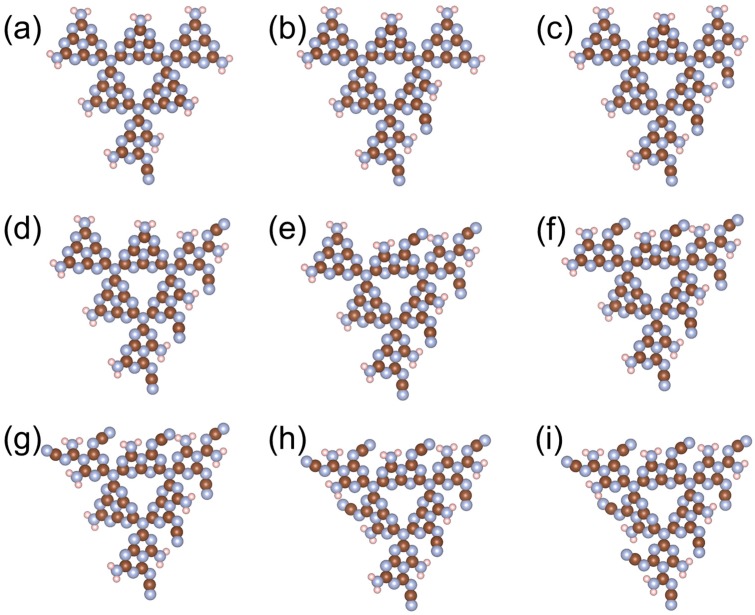
Structures of g-C_3_N_4_-6 with varying numbers of C≡N groups. Brown, blue, and pink spheres represent C, N, and H, respectively.

**Figure 4:**
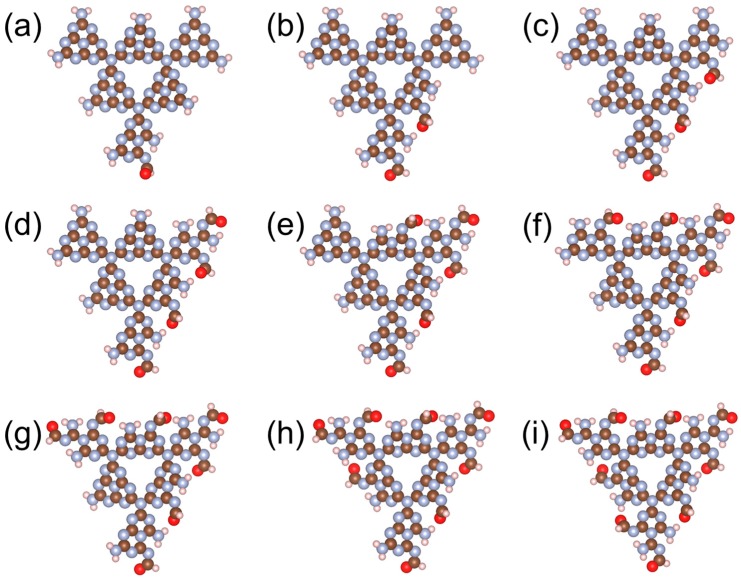
Structures of g-C_3_N_4_-6 with varying numbers of C=O groups. Brown, blue, pink, and red spheres represent C, N, H, and O, respectively.

**Figure 5:**
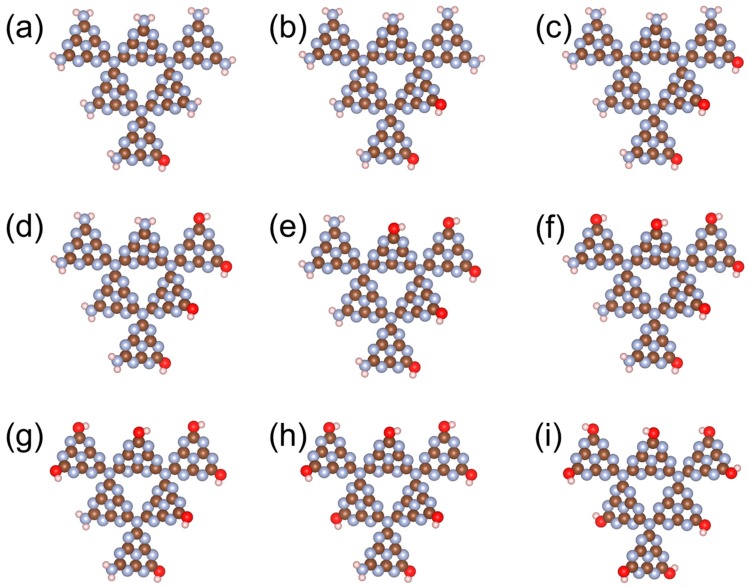
Structures of g-C_3_N_4_-6 with varying numbers of O−H groups. Brown, blue, pink, and red spheres represent C, N, H, and O, respectively.

With the conclusion from Figure 1, that a g-C_3_N_4_ structure with six repeated g-C_3_N_4_ units (g-C_3_N_4_-6) is large enough to represent a periodic structure, all further calculations were performed with this critical size. Figure 2 shows the tuning of the HOMO−LUMO gap with the increasing number of C≡N, C=O, and O−H in a g-C_3_N_4_-6 structure (the structural information can be found in Figure 3, Figure 4 and Figure 5). Interestingly, although all of the three trends are not monotonic, they generally show that the existence of C≡N and C=O can significantly narrow down the HOMO–LUMO gap, while O−H can slightly raise the gap. The differences on the effects of functional groups might originate from the different electronic properties among the functional groups: O−H is electron-donating, while C≡N and C=O are electron-withdrawing. Tian et al. [21] suggested that substitutes with electron-donating and -withdrawing properties could lead to the different distribution of HOMO and LUMO. In this study, our results suggest that the form of carbon and oxygen contained in the g-C_3_N_4_ are particularly important: for a g-C_3_N_4_ structure, a certain ratio of C≡N and C=O may narrow down the energy band gap to the optimized value, leading to higher photocatalytic performance. From an experimental perspective, it is expected that the preparation of g-C_3_N_4_ substituted with more electron-withdrawing groups could be beneficial to both scientific and industrial applications.

## 4. Conclusions

In this communication, we have shown the size and functional group effects on g-C_3_N_4_ using DFT calculations. It was found that a g-C_3_N_4_-6 molecule could be the smallest unit that converges to the limit of the HOMO–LUMO gap. Calculations of g-C_3_N_4_-6 with varying numbers of substituted C≡N, C=O, and O−H functional groups have shown that generally, C≡N and C=O could narrow down the HOMO–LUMO gap, while O−H could slightly raise the gap. This study shows that rationally modifying the substituent at the edge of g-C_3_N_4_-based materials during band gap engineering could help to increase the catalytic performance. In future studies, we will focus on revealing more physical understanding behind these functional group effects.
